# Structural properties and asymptotic behavior of bacterial two-component systems

**DOI:** 10.3389/fsysb.2025.1693064

**Published:** 2025-10-21

**Authors:** Irene Zorzan, Chiara Cimolato, Luca Schenato, Massimo Bellato

**Affiliations:** ^1^ Department of Information Engineering, Università degli Studi di Padova, Padova, Italy; ^2^ Department of Molecular Medicine, Università degli Studi di Padova, Padova, Italy

**Keywords:** two-component systems, MprAB *Mycobacterium*, EnvZ, OmpR, synthetic biology, sensor histidine kinase, response regulator, odes

## Abstract

Bacteria rely on two-component signaling systems (TCSs) to detect environmental cues and orchestrate adaptive responses. Despite their apparent simplicity, TCSs exhibit a rich spectrum of dynamic behaviors arising from network architectures, such as bifunctional enzymes, multi-step phosphorelays, transcriptional feedback loops, and auxiliary interactions. This study develops a generalized mathematical model of a TCS that integrates these various elements. Using systems-level analysis, we elucidate how network architecture and biochemical parameters shape key properties such as stability, monotonicity, and signal amplification. Analytical conditions are derived for when the steady-state levels of phosphorylated proteins exhibit robustness to variations in protein abundance. The model characterizes how equilibrium phosphorylation levels depend on the absolute and relative abundances of the two components. Specific scenarios are explored, including the MprAB system from *Mycobacterium tuberculosis* and the EnvZ/OmpR system from textit *Escherichia coli*, to describe the potential role of reverse phosphotransfer reactions. By combining mechanistic modeling with system-level techniques, such as nullcline analysis, this study offers a unified perspective on the design principles underlying the versatility of bacterial signal transduction. The generalized modeling framework lays a theoretical foundation for interpreting experimental dynamics and rationally engineering synthetic TCS circuits with prescribed response dynamics.

## 1 Introduction

Bacteria rely on two-component systems (TCSs) as their primary signaling modules to detect environmental cues and orchestrate adaptive responses. A canonical TCS consists of a membrane-bound sensor histidine kinase (SHK) and a cytoplasmic response regulator (RR). Upon stimulation, the SHK autophosphorylates on a conserved histidine and transfers the phosphoryl group to an aspartate on the RR, generating the active form (RR-P) that typically regulates gene expression. This minimal architecture is remarkably versatile, underpinning processes such as chemotaxis, nutrient sensing, antibiotic resistance, and virulence regulation (Tierney and Rather, 2019; Tiwari et al., 2017; Kirby, 2009; Ramos et al., 2022; Alvarez and Georgellis, 2023).

Despite their apparent simplicity, TCSs display a rich spectrum of topologies and dynamic behaviors (Zschiedrich et al., 2016; Groisman, 2016; Stock et al., 2000). In some systems, exemplified by CheA in bacterial chemotaxis, SHK functions exclusively as a kinase, phosphorylating the RR. However, in many TCSs, SHK is bifunctional, participating in both phosphorylation and dephosphorylation of its cognate RR. In such cases, the input signal can modulate either one or both of these enzymatic activities, effectively tuning the rates of kinase and/or phosphatase reactions. TCSs may implement single-step phosphotransfers or multi-step phosphorelays, adding regulatory complexity and potentially delaying signal propagation.

At the transcriptional level, many TCSs feature autoregulation: the phosphorylated RR activates transcription of both its own gene and the gene encoding its partner SHK, thereby forming a positive feedback loop (Goulian, 2010). This feedback can alter steady-state behavior, activation, and inactivation kinetics and generate transient overshoot or “memory” effects, whereby the system responds faster to repeated stimuli. Although less common, negative autoregulation—or even mixed positive and negative feedback—has been observed in specific systems, providing an additional layer of response modulation. Auxiliary proteins can further diversify TCS behaviors, either by directly interacting with SHKs or RRs or by mediating cross-talk between otherwise independent TCS pathways (Rao et al., 2021; Groisman, 2016).

Mathematical modeling has been pivotal in elucidating the emergent properties of TCSs (summarized in Table 1). Batchelor and Goulian (2003) demonstrated that the steady-state level of RR-P can be robust to protein abundance fluctuations when SHK is limiting, a property supported by experimental data. Shinar et al. (2007) formalized the conditions for input-output robustness, showing that robustness is compromised when multiple independent phosphorylation or dephosphorylation routes exist. Igoshin et al. (2008) identified conditions for bistability, particularly when unphosphorylated SHK and RR form “dead-end” complexes or when alternative phosphatases modulate RR-P turnover. Ray and Igoshin (2010), Mitrophanov et al. (2010), and Zorzan et al. (2021) explored the role of transcriptional feedback, showing that autoregulation can alter response speed, overshoot amplitude, and even affect the effective sign of feedback, enabling TCSs to switch between positive and negative regulatory modes depending on signal strength. These studies collectively highlight how bifunctionality, phosphorelays, and feedback loops produce rich dynamic behaviors—including robustness, bistability, and adaptive memory—that are now central themes in systems-level analyses of TCSs.

**TABLE 1:** Comparison of previous findings on bacterial TCSs with results from this study’s model.

References	Findings from previous studies	Model results of this study
Batchelor and Goulian (2003)	Robustness of RR-P steady-state levels when SHK is limiting; EnvZ/OmpR experiments confirmed robustness to fluctuations in protein abundance.	Reproduces robustness when exogenous phosphorylation is absent. Predicts loss of robustness (steady state depends on SHK:RR ratio) if exogenous phosphorylation flux is present.
Shinar et al. (2007)	Formalized conditions for input–output robustness; robustness breaks down when multiple phosphorylation/dephosphorylation pathways exist.	General model confirms robustness only under restricted architectures. Multiple independent routes compromise robustness.
(Dutta and Inouye, 1996); (Zhu et al., 2000)	Proposed and observed reverse phosphotransfer (RR-P → SHK) in EnvZ/OmpR; debated as mechanism for phosphatase activity.	Extends framework to include reverse phosphotransfer. Predicts that it does not affect RR-P steady state (compensated by forward transfer), but increases phosphorylated SHK levels.

In this study, we develop a systems-level model of a generalized TCS model focusing on the MprAB system from *Mycobacterium tuberculosis* that integrates canonical phosphorylation cycles, bifunctional enzymatic activity, transcriptional feedback, and potential auxiliary interactions. Our modeling framework seeks to (i) dissect how network architecture and parameter regimes shape dynamic properties and provide robustness, to be adopted as a building block to implement overshoots, oscillations, and bistability, and (ii) provide a predictive foundation for interpreting experimental dynamics and guiding synthetic circuit design in bacterial signal transduction.

By combining mechanistic modeling with systems-level analysis, this study elucidates how bifunctionality, phosphorelays, and feedback loops shape the dynamic behavior of TCSs, providing insights into bacterial adaptation and a framework for the rational engineering of synthetic signaling circuits (Mukherji and van Oudenaarden, 2009; Pasotti et al., 2017; Müller et al., 2025).

## 2 Two-component system: mathematical model

The model we consider is a general version of the model proposed in Tiwari et al. (2010) to describe the functioning of the two-component system *MprA/MprB* in *M. tuberculosis* in its active state.

For the sake of generality, we refer to “response regulator” (RR) and “sensor histidine kinase” (SHK) rather than to *MprA* and *MprB*, respectively. Denoting by r $\left(r^{*}\right)$ and s $\left(s^{*}\right)$ , the concentration of RR (phosphorylated RR ) and SHK (phosphorylated SHK ), respectively, the dynamic evolution of the two-component system is described by the following set of ODEs (see Supplemental Information of Tiwari et al. (2010), Equations (S39)–(S42)):

$$\dot{r}=\frac{k_{p}}{K_{P}}r^{*}s-\frac{k_{t}}{K_{T}}rs^{*}+k_{\mathrm{exd}}r^{*}-k_{\exp}r+\nu_{r}-k_{\mathrm{pdeg}}r$$ (1)

$${\dot{r}}^{*}=-\frac{k_{p}}{K_{P}}r^{*}s+\frac{k_{t}}{K_{T}}rs^{*}-k_{\mathrm{exd}}r^{*}+k_{\exp}r-k_{\mathrm{pdeg}}r^{*}$$ (2)

$$\dot{s}=k_{ad}s^{*}-k_{ap}s+\frac{k_{t}}{K_{T}}rs^{*}+\nu_{s}-k_{\mathrm{pdeg}}s$$ (3)

$${\dot{s}}^{*}=-k_{ad}s^{*}+k_{ap}s-\frac{k_{t}}{K_{T}}rs^{*}-k_{\mathrm{pdeg}}s^{*}$$ (4)

—where

• $\nu_{r}$ and $\nu_{s}$ are the production rate constants of RR and SHK , respectively;^1^

• $k_{p}$ is the rate constant for the SHK -dependent dephosphorylation of $RR^{*}$ ;

• $K_{P}$ is the Michaelis–Menten constant for $RR^{*}$ dephosphorylation by SHK ;

• $k_{t}$ is the rate constant for the $SHK^{*}$ -dependent phosphorylation of RR ;

• $K_{T}$ is the Michaelis–Menten constant for RR – $SHK^{*}$ phosphotransfer;

• $k_{\exp}$ and $k_{\mathrm{exd}}$ are the exogenous phosphorylation and dephosphorylation rate constants, respectively;

• $k_{ap}$ and $k_{ad}$ are the autophopshorylation and autodephosphorylation rate constants, respectively;

• $k_{\mathrm{pdeg}}$ is the protein degradation rate (assumed equal for RR and SHK ).

One additional assumption worth highlighting is that the system is always considered to be in the active state. This is biologically reasonable as external stimuli often saturate the sensing capacity of the TCS. As a result, the transition of the sensor s from the inactive to the active state upon binding external stimuli can be neglected in the model, as well as the availability of ATP inside the cell to provide phosphate groups for the phosphorylation steps.

**FIGURE 1:**
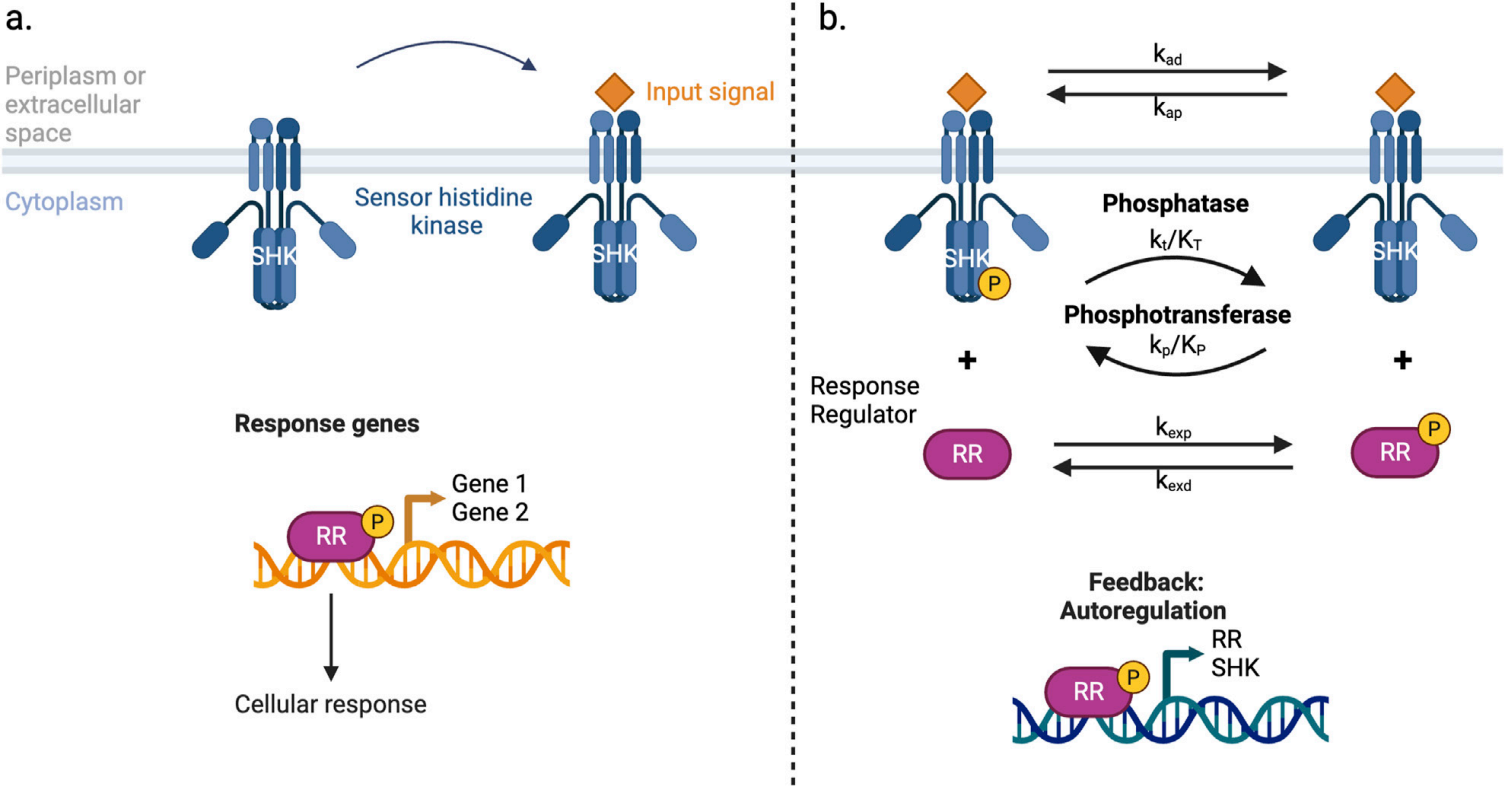
Schema of the generalized TCS. Binding of the signal molecule and general activation of genes are reported in panel **(a)**, while in panel **(b)** the part of the system described by Equations 1–4 is reported.

The overall system can be represented as in Figure 1.

We define the total amount of RR and SHK as $R_{T}=r+r^{*}$ and $S_{T}=s+s^{*}$ , respectively, and rewrite the previous model presented in Equations 1–4 in the form shown in Equations 5–8:

$${\dot{R}}_{T}=k_{\mathrm{pdeg}}\left(u_{r}-R_{T}\right)$$ (5)

$${\dot{r}}^{*}=-\frac{k_{p}}{K_{P}}r^{*}\left(S_{T}-s^{*}\right)+\frac{k_{t}}{K_{T}}\left(R_{T}-r^{*}\right)s^{*}-k_{\mathrm{exd}}r^{*}+k_{\exp}\left(R_{T}-r^{*}\right)-k_{\mathrm{pdeg}}r^{*}$$ (6)

$${\dot{S}}_{T}=k_{\mathrm{pdeg}}\left(u_{s}-S_{T}\right)$$ (7)

$${\dot{s}}^{*}=-k_{ad}s^{*}+k_{ap}\left(S_{T}-s^{*}\right)-\frac{k_{t}}{K_{T}}\left(R_{T}-r^{*}\right)s^{*}-k_{\mathrm{pdeg}}s^{*}$$ (8)

—where $u_{r}≔\nu_{r}/k_{\mathrm{pdeg}}$ $\left(u_{s}≔\nu_{s}/k_{\mathrm{pdeg}}\right)$ is the net production rate of RR (SHK) . Due to the separation of timescales between protein accumulation and phosphorylation/dephosphorylation events, we can assume that total concentrations of RR and SHK are preserved—namely, that $R_{T}$ and $S_{T}$ are constant. Under this assumption, we can normalize all state variables and consider the phosphorylated portion of RR and SHK

$$r^{*}≔\frac{r^{*}}{R_{T}}\;\text{and}\;s^{*}≔\frac{s^{*}}{S_{T}},$$

the dynamics of which are described by

$$\begin{array}{ll} {\dot{r}}^{*} & =-\left(k_{\mathrm{exd}}+k_{\exp}+k_{\mathrm{pdeg}}\right)r^{*}-\frac{k_{p}}{K_{P}}S_{T}r^{*}\left(1-s^{*}\right)-\frac{k_{t}}{K_{T}}S_{T}r^{*}s^{*}+\frac{k_{t}}{K_{T}}S_{T}s^{*}+k_{\exp}\\ {\dot{s}}^{*} & =-\left(k_{ad}+k_{ap}+k_{\mathrm{pdeg}}\right)s^{*}-\frac{k_{t}}{K_{T}}R_{T}s^{*}\left(1-r^{*}\right)+k_{ap} \end{array}$$

Since we aim to provide a model describing the functioning of *general* two-component systems (TCSs) and unveiling its structural and asymptotic properties, from now on we will consider the following general formulation:

$${\dot{r}}^{*}=-\left(\alpha_{1}+\alpha_{2}\right)r^{*}-\alpha_{3}S_{T}r^{*}\left(1-s^{*}\right)-\alpha_{4}S_{T}r^{*}s^{*}+\alpha_{4}S_{T}s^{*}+\alpha_{2}≕f_{1}\left(r^{*},s^{*}\right)$$ (9)

$${\dot{s}}^{*}=-\left(\beta_{1}+\beta_{2}\right)s^{*}-\beta_{3}R_{T}s^{*}\left(1-r^{*}\right)-\beta_{4}R_{T}r^{*}s^{*}+\beta_{4}R_{T}r^{*}+\beta_{2}≕f_{2}\left(r^{*},s^{*}\right)$$ (10)

Differential Equations 9, 10 describe the dynamics of the phosphorylated portions of RR and SHK —that is, ratio phosphorylated- RR (phosphorylated- SHK ) over total RR (SHK) —under the assumption that total concentrations $R_{T}$ and $S_{T}$ are constant. Notice that in Equation 10, the terms $-\beta_{4}R_{T}r^{*}s^{*}$ and $\beta_{4}R_{T}r^{*}$ have been included for reasons of symmetry. Of course, this general formulation can be tailored to the specific two-component system under investigation. For instance, we immediately verify that, upon defining

$$\begin{array}{llllllll} \alpha_{1} & =k_{\mathrm{exd}}+k_{\mathrm{pdeg}}, & \alpha_{2} & =k_{\exp}, & \alpha_{3} & =\frac{k_{p}}{K_{P}}, & \alpha_{4} & =\frac{k_{t}}{K_{T}}\\ \beta_{1} & =k_{ad}+k_{\mathrm{pdeg}}, & \beta_{2} & =k_{ap}, & \beta_{3} & =\frac{k_{t}}{K_{T}}, & \beta_{4} & =0, \end{array}$$

Equations 9, 10 reduce to the *MprA*-*MprB* system proposed in Tiwari et al. (2010).

### 2.1 Structural properties

We note that, by the way that $r^{*}$ has been defined, it is dimensionless, and such that for every t≥0 it holds $0\leq r^{*}\left(t\right)\leq 1$ , $r^{*}=0$ means that all RR are unphosphorylated, while $r^{*}=1$ represents the situation with all RR phosphorylated. Clearly, the same holds for $s^{*}$ , and hence every state trajectory of the bidimensional system Equations 9, 10 belongs to the feasibility set $C≔\left\{\left(r^{*},s^{*}\right):0\leq r^{*}\leq 1,\;0\leq s^{*}\leq 1\right\}$ .

Proposition 1 *The TCS model* Equations 9, 10 *exhibits a unique equilibrium point* $\left(r_{eq}^{*},s_{eq}^{*}\right)$ *within the feasibility set* C .

Proof. First, notice that the set C is positively invariant with respect to systems Equations 9, 10, so that if the state trajectory starts in C , then it stays in C for any t≥0 . Positive invariance of the convex and compact set C ensures that there exists at least one equilibrium point in C —that is, a limit cycle or at least one stable equilibrium point (Blanchini and Miani, 2015—Theorem 4.21).

We now resort to Bendixon’s theorem to rule out the existence of closed orbits.^2^ Note that

$$\begin{array}{llll} \frac{df_{1}}{dr^{*}}\left(r^{*},s^{*}\right) & =-\left(\alpha_{1}+\alpha_{2}\right)-\alpha_{3}S_{T}\left(1-s^{*}\right)-\alpha_{4}S_{T}s^{*}<0, & & \forall \left(r^{*},s^{*}\right)\in C\\ \frac{df_{2}}{ds^{*}}\left(r^{*},s^{*}\right) & =-\left(\beta_{1}+\beta_{2}\right)-\beta_{3}R_{T}\left(1-r^{*}\right)-\beta_{4}R_{T}r^{*}<0, & & \forall \left(r^{*},s^{*}\right)\in C \end{array}$$

Hence, $div\left(f\right)≔\frac{df_{1}}{dr^{*}}+\frac{df_{2}}{ds^{*}}$ is not identically zero in any sub-region of the simply connected region C and does not change sign in C . Then, by Bendixon’s theorem (Sastry, 1999—Theorem 2.7), the set C contains no closed orbits of system Equations 9, 10.

Finally, we resort to nullcline analysis to prove the uniqueness of steady states. Setting $dr^{*}/dt=0$ and $ds^{*}/dt=0$ yields the following expressions for $r^{*}$ and $s^{*}$ nullclines:

$$r^{*}=\frac{\alpha_{4}S_{T}s^{*}+\alpha_{2}}{\alpha_{1}+\alpha_{2}+\alpha_{3}S_{T}\left(1-s^{*}\right)+\alpha_{4}S_{T}s^{*}}≕g\left(s^{*}\right)$$ (11)

$$s^{*}=\frac{\beta_{4}R_{T}r^{*}+\beta_{2}}{\beta_{1}+\beta_{2}+\beta_{3}R_{T}\left(1-r^{*}\right)+\beta_{4}R_{T}r^{*}}≕h\left(r^{*}\right)$$ (12)

**FIGURE 2:**
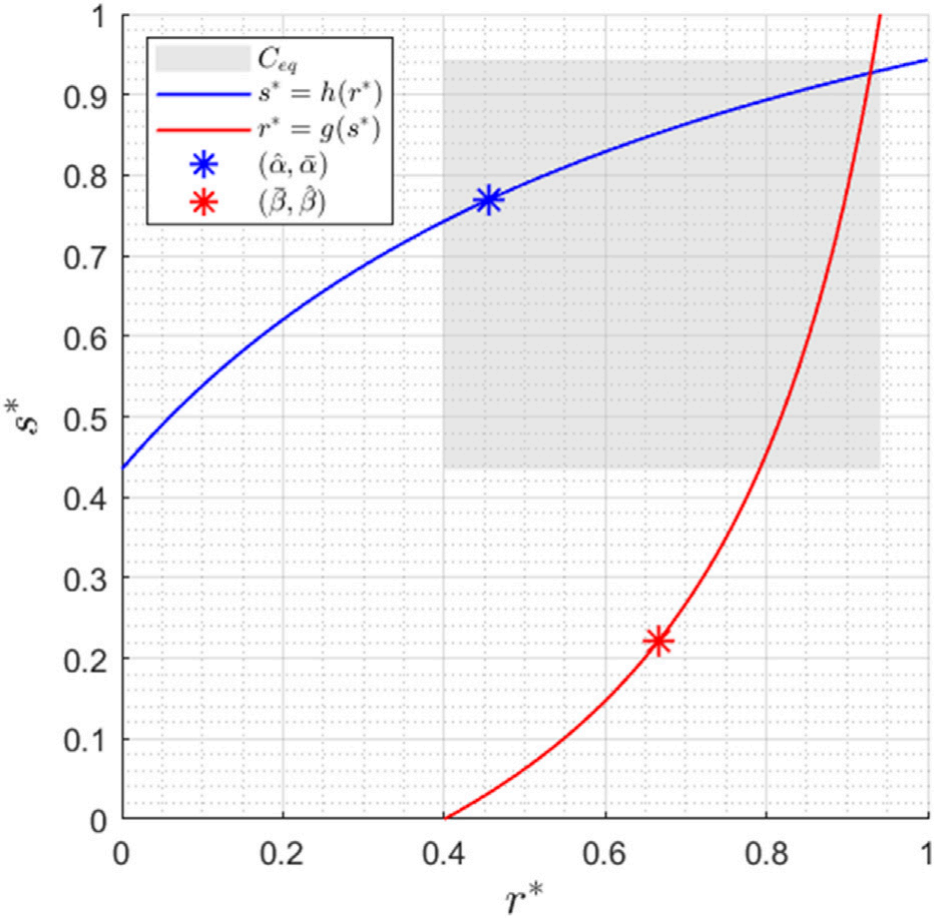
Nullclines for $\alpha_{1}=0.5$ , $\alpha_{2}=1$ , $\alpha_{3}=1$ , $\alpha_{4}=7$ , $\beta_{1}=0.3$ , $\beta_{2}=1$ , $\beta_{3}=1$ , $\beta_{4}=4$ , $R_{T}=1$ , and $S_{T}=1$ . The $C_{eq}$ region corresponds to the subregion where the equilibrium point is located, as detailed in Proposition 2.

A typical figure of RR and SHK nullclines is reported in Figure 2.

From expression 11, it is easy to obtain $s^{*}=g^{-1}\left(r^{*}\right)$ :

$$g^{-1}\left(r^{*}\right)=\frac{\left(\alpha_{1}+\alpha_{2}+\alpha_{3}S_{T}\right)r^{*}-\alpha_{2}}{\alpha_{4}S_{T}\left(1-r^{*}\right)+\alpha_{3}S_{T}r^{*}}$$ (13)

We define the function $\Delta \left(r^{*}\right)≔h\left(r^{*}\right)-g^{-1}\left(r^{*}\right)$ and note that, by the way $\Delta \left(r^{*}\right)$ has been defined, if $\left(r_{eq}^{*},s_{eq}^{*}\right)$ is an equilibrium point, then $\Delta \left(r_{eq}^{*}\right)=0$ ; *vice versa*, if $\Delta \left({\bar{r}}^{*}\right)=0$ then $\left({\bar{r}}^{*},h\left({\bar{r}}^{*}\right)\right)=\left(r_{eq}^{*},s_{eq}^{*}\right)$ is an equilibrium point. It is a matter of computation to verify that $\Delta \left(r^{*}\right)$ is a rational function— $\Delta \left(r^{*}\right)=\frac{n\left(r^{*}\right)}{d\left(r^{*}\right)}$ —and that both the numerator and denominator are polynomials of order 2:

$$\begin{array}{ll} n\left(r^{*}\right)= & \left(\beta_{4}R_{T}r^{*}+\beta_{2}\right)\left(\alpha_{4}S_{T}\left(1-r^{*}\right)+\alpha_{3}S_{T}r^{*}\right)\\ & +-\left(\beta_{1}+\beta_{2}+\beta_{3}R_{T}\left(1-r^{*}\right)+\beta_{4}R_{T}r^{*}\right)\\ & \times \left(\left(\alpha_{1}+\alpha_{2}+\alpha_{3}S_{T}\right)r^{*}-\alpha_{2}\right) \end{array}$$

$$\begin{array}{ll} d\left(r^{*}\right) & =\left(\beta_{1}+\beta_{2}+\beta_{3}R_{T}\left(1-r^{*}\right)+\beta_{4}R_{T}r^{*}\right)\left(\alpha_{4}S_{T}\left(1-r^{*}\right)+\alpha_{3}S_{T}r^{*}\right) \end{array}$$

Note that $d\left(r^{*}\right)>0$ for every $r^{*}\in \left[0,1\right]$ , and hence $\Delta \left(r^{*}\right)=0$ for some $r^{*}\in \left[0,1\right]$ if and only if $n\left(r^{*}\right)=0$ for some $r^{*}\in \left[0,1\right]$ . Since n(0)>0 and n(1)<0 , there certainly exists $r_{eq}^{*}\in \left[0,1\right]$ such that $n\left(r_{eq}^{*}\right)=0$ , and hence $\Delta \left(r_{eq}^{*}\right)=0$ —as already demonstrated, the system admits at least one equilibrium point in C . On the other hand, since $n\left(r^{*}\right)$ is a second-order polynomial, such an $r_{eq}^{*}$ belonging to the interval [0,1] is unique—the system admits a unique equilibrium point C .

Remark 1 Remark 1. A closed-form expression for the equilibrium point of the TCS can be computed as the unique root in interval [0,1] of the second-order polynomial $n\left(r^{*}\right)$ *.*

$$\begin{array}{ll} r_{eq}^{*} & =\alpha_{3}\beta_{3}R_{T}S_{T}-\alpha_{4}\beta_{4}R_{T}S_{T}\pm \\ & \frac{\sqrt{A}}{\left(2\left(\alpha_{3}\beta_{3}R_{T}S_{T}-\alpha_{4}\beta_{4}R_{T}S_{T}+\alpha_{1}\beta_{3}R_{T}+\alpha_{2}\beta_{3}R_{T}-\alpha_{1}\beta_{4}R_{T}-\alpha_{2}\beta_{4}R_{T}\right)\right)} \end{array}$$

with. $\begin{array}{l} A=(-\alpha_{3}\beta_{3}R_{T}S_{T}+\alpha_{4}\beta_{4}R_{T}S_{T}-\alpha_{1}\beta_{3}R_{T}-2\alpha_{2}\beta_{3}R_{T}+\alpha_{2}\beta_{4}R_{T}-\alpha_{3}\beta_{1}S_{T}-\alpha_{4}\beta_{2}S_{T}\\ {-\alpha_{1}\beta_{1}-\alpha_{2}\beta_{1}-\alpha_{1}\beta_{2}-\alpha_{2}\beta_{2})}^{2}-4\left(\alpha_{2}\beta_{3}R_{T}+\alpha_{4}\beta_{2}S_{T}+\alpha_{2}\beta_{1}+\alpha_{2}\beta_{2}\right)\\ (\alpha_{3}\beta_{3}R_{T}S_{T}-\alpha_{4}\beta_{4}R_{T}S_{T}+\alpha_{1}\beta_{3}R_{T}+\alpha_{2}\beta_{3}R_{T}-\alpha_{1}\beta_{4}R_{T}-\alpha_{2}\beta_{4}R_{T}\\ )+\alpha_{1}\beta_{3}R_{T}+2\alpha_{2}\beta_{3}R_{T}-\alpha_{2}\beta_{4}R_{T}+\alpha_{3}\beta_{1}S_{T}\\ +\alpha_{4}\beta_{2}S_{T}+\alpha_{1}\beta_{1}+\alpha_{2}\beta_{1}+\alpha_{1}\beta_{2}+\left.\alpha_{2}\beta_{2}\right) \end{array}$

Proposition 1 states that all trajectories with initial conditions in C converge to a unique equilibrium point $\left(r_{eq}^{*},s_{eq}^{*}\right)\in C$ . This means that, independently of the initial relative amounts of phosphorylated and unphosphorylated proteins, the proportion of phosphorylated to total RR will asymptotically equal $r_{eq}^{*}$ , while the proportion of phosphorylated to total SHK will asymptotically tend to $s_{eq}^{*}$ . The following proposition identifies a subregion $C_{eq}⊊C$ where the equilibrium point is located and hence provides upper and lower bounds to the phosphorylation levels $r_{eq}^{*}$ and $s_{eq}^{*}$ asymptotically reached by the TCS.

Proposition 2 Consider the TCS described by models Equations 9, 10. The unique equilibrium point of the system, denoted by $\left(r_{eq}^{*},s_{eq}^{*}\right)$ *, belongs to the subregion*

$$C_{eq}≔\left\{\left(r^{*},s^{*}\right):r_{\min}^{*}\leq r^{*}\leq r_{\max}^{*},\;s_{\min}^{*}\leq s^{*}\leq s_{\max}^{*}\right\}⊊C,$$

where

$$\begin{array}{llll} r_{\min}^{*}: & =\frac{\alpha_{2}}{\alpha_{1}+\alpha_{2}+\alpha_{3}S_{T}}, & r_{\max}^{*}: & =\frac{\alpha_{4}S_{T}+\alpha_{2}}{\alpha_{1}+\alpha_{2}+\alpha_{4}S_{T}},\\ s_{\min}^{*}: & =\frac{\beta_{2}}{\beta_{1}+\beta_{2}+\beta_{3}R_{T}}, & s_{\max}^{*}: & =\frac{\beta_{4}R_{T}+\beta_{2}}{\beta_{1}+\beta_{2}+\beta_{4}R_{T}} \end{array}$$

Proof. Consider the expression for RR nullcline Equation 11 and note that

$$\frac{\partial g}{\partial s^{*}}\left(s^{*}\right)=\frac{S_{T}\left(\alpha_{1}\alpha_{4}+\alpha_{4}\alpha_{3}S_{T}+\alpha_{2}\alpha_{3}\right)}{{\left(\alpha_{1}+\alpha_{2}+\alpha_{3}S_{T}\left(1-s^{*}\right)+\alpha_{4}S_{T}s^{*}\right)}^{2}}>0\;\text{for every}s^{*}\in \left[0,1\right],$$

and hence $r^{*}$ is strictly monotonically increasing in $s^{*}$ . The bounds on $r_{eq}^{*}$ then follow from

$$g\left(0\right)=\frac{\alpha_{2}}{\alpha_{1}+\alpha_{2}+\alpha_{3}S_{T}}≕r_{\min}^{*},\;\text{and}\;g\left(1\right)=\frac{\alpha_{4}S_{T}+\alpha_{2}}{\alpha_{1}+\alpha_{2}+\alpha_{4}S_{T}}≕r_{\max}^{*},$$

Analogous computations on SHK nullcline Equation 12 lead to upper and lower bounds on $s_{eq}^{*}$ .

The set $C_{eq}$ is reported in Figure 2 for the set of parameters considered. We conclude this section with the following Lemma, which will be useful for subsequent derivations (see again Figure 2).

Lemma 1 *Consider the TCS described by models* Equations 9, 10, *and define*

$$\begin{array}{llll} \bar{\alpha }: & =\frac{\alpha_{2}\alpha_{3}}{\alpha_{2}\alpha_{3}+\alpha_{1}\alpha_{4}} & \hat{\alpha }: & =\frac{\alpha_{2}}{\alpha_{1}+\alpha_{2}}\\ \bar{\beta }: & =\frac{\beta_{2}\beta_{3}}{\beta_{2}\beta_{3}+\beta_{1}\beta_{4}} & \hat{\beta }: & =\frac{\beta_{2}}{\beta_{1}+\beta_{2}} \end{array}$$

*Then,* RR *nullcline* Equation 11 *always passes through* $\left(\bar{\alpha },\hat{\alpha }\right)$ — $g\left(\bar{\alpha }\right)=\hat{\alpha }$ —*while* SHK *nullcline* Equation 12 *always passes through* $\left(\bar{\beta },\hat{\beta }\right)$ — $h\left(\bar{\beta }\right)=\hat{\beta }$ .

**FIGURE 3:**
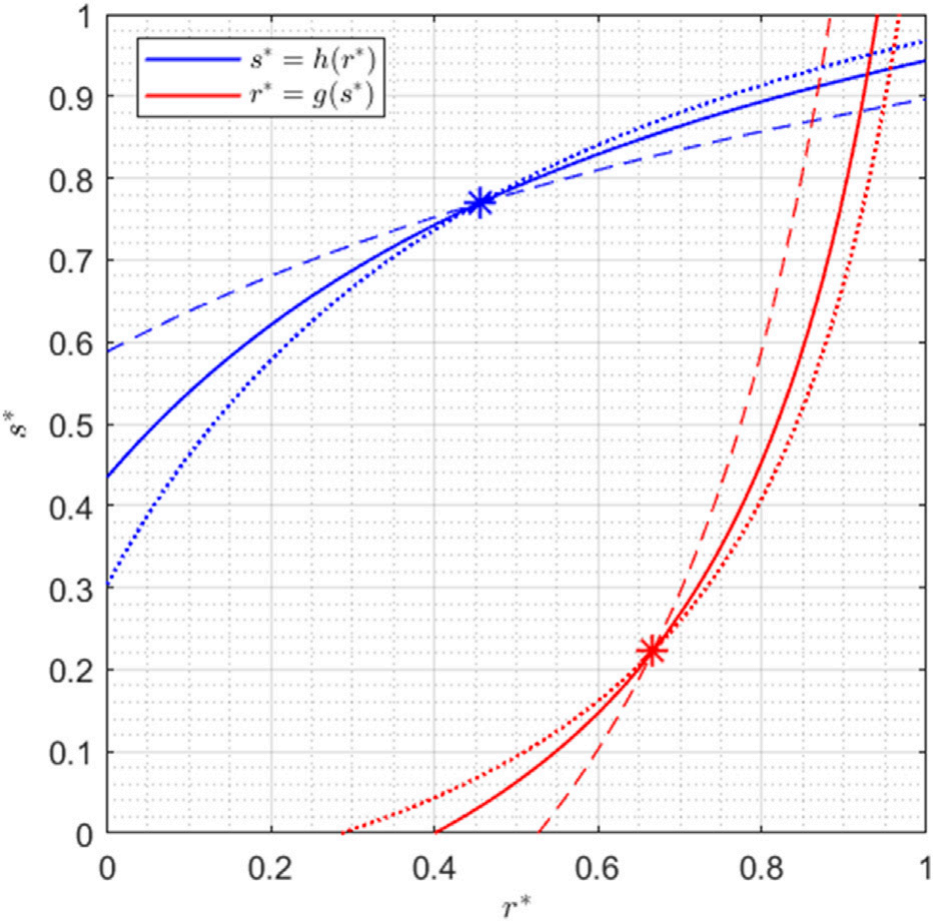
Nullclines for $\alpha_{1}=0.5$ , $\alpha_{2}=1$ , $\alpha_{3}=1$ , $\alpha_{4}=7$ , $\beta_{1}=0.3$ , $\beta_{2}=1$ , $\beta_{3}=1$ , and $\beta_{4}=4$ . The solid lines have $R_{T}=1$ and $S_{T}=1$ as in Figure 2; the dashed lines are obtained with $R_{T}=0.4$ and $S_{T}=0.4$ ; the dotted lines with $R_{T}=2$ and $S_{T}=2$ .

This behavior can also be observed in Figure 3, where the dotted lines indicate the nullclines associated with higher values of $R_{T}$ and $S_{T}$ , while the dashed lines are the nullclines obtained with lower values of $R_{T}$ and $S_{T}$ , as described in the caption.

Since verifying that $g\left(\bar{\alpha }\right)=\hat{\alpha }$ and $h\left(\bar{\beta }\right)=\hat{\beta }$ is just a matter of computation, the proof of Lemma 1 is omitted.

At this point, two observations are in order. First, the dimensionless values $r_{eq}^{*}$ and $s_{eq}^{*}$ depend on the total amounts of RR and SHK proteins present within the system (recall that, due to time scale separation, so far we have assumed that the quantities $R_{T}$ and $S_{T}$ are constant). In other words, $r_{eq}^{*}$ and $s_{eq}^{*}$ are continuous functions of $R_{T}$ and $S_{T}$ — $r_{eq}^{*}=r_{eq}^{*}\left(R_{T},S_{T}\right)$ and $s_{eq}^{*}=s_{eq}^{*}\left(R_{T},S_{T}\right)$ . The second observation is that uniform monotonicity of $r_{eq}^{*}\left(R_{T},S_{T}\right)$ and $s_{eq}^{*}\left(R_{T},S_{T}\right)$ with respect to their arguments is not guaranteed. Depending on the values taken by the system parameters, equilibrium $r_{eq}^{*}$ might decrease with $R_{T}$ when $R_{T}$ belongs to a specific interval, and increase with $R_{T}$ when it belongs to a different interval.

## 3 Relative concentrations

### 3.1 Low vs high $R_{T}$ concentration

In this section, we assume that $R_{T}$ and $S_{T}$ are independent.

Proposition 3 *(Low* $R_{T}$ *concentration.) Consider the TCS described by models* Equations 9, 10 *and let the total* SHK *concentration* $S_{T}$ *be arbitrary but fixed. When the total* RR *concentration is extremely low—that is, for* $R_{T}\to 0$ *—the equilibrium point asymptotically reached by the system is given by* $\left(r_{eq}^{*},s_{eq}^{*}\right)=\left(g\left(\hat{\beta }\right),\hat{\beta }\right)$ .

Proof. By taking the limit for $R_{T}\to 0$ of the function $h\left(r^{*}\right)$ defined in Equation 12 and representing SHK nullcline,^3^ it can be seen that $s_{eq}^{*}=\hat{\beta }$ . The result then follows by plugging $s_{eq}^{*}$ into RR -nullcine Equation 11.

Proposition 4 (High $R_{T}$ concentration.) Consider the TCS described by models Equations 9, 10 and let the total SHK concentration $S_{T}$ be arbitrary but fixed. When the total RR concentration is extremely high—that is, for $R_{T}\to +\infty$ —the equilibrium point asymptotically reached by the system is $\left(r_{hR}^{*},h\left(r_{hR}^{*}\right)\right)$ , with $r_{hR}^{*}$ being the (unique) solution in the interval [0,1] of the quadratic equation $A{\left(r^{*}\right)}^{2}+Br^{*}+C=0$ , where

$$\begin{array}{ll} A: & =\left(\alpha_{1}+\alpha_{2}+\alpha_{3}S_{T}\right)\beta_{3}-\left(\alpha_{2}+\alpha_{4}S_{T}\right)\beta_{4}-\alpha_{1}\beta_{4}\\ B: & =-\left(\alpha_{1}+\alpha_{2}+\alpha_{3}S_{T}\right)\beta_{3}+\left(\alpha_{2}+\alpha_{4}S_{T}\right)\beta_{4}-\alpha_{2}\beta_{3}\\ C: & =\alpha_{2}\beta_{3} \end{array}$$

More specifically, $r_{hR}^{*}=\left(-B-\sqrt{B^{2}-4AC}\right)/\left(2A\right)$ .

Proof. Note that when $R_{T}\to +\infty$ , the upper and lower bounds on $s_{eq}^{*}$ are given by $s_{\min}^{*}=0$ and $s_{\max}^{*}=1$ , respectively, and hence do not provide any useful information. Taking the limit for $R_{T}\to +\infty$ of RR and SHK nullclines Equations 13, 12 yields

$$\begin{array}{ll} \underset{R_{T}\to +\infty }{\lim}g^{-1}\left(r^{*}\right) & =\frac{\left(\alpha_{1}+\alpha_{2}+\alpha_{3}S_{T}\right)r^{*}-\alpha_{2}}{\alpha_{4}S_{T}\left(1-r^{*}\right)+\alpha_{3}S_{T}r^{*}}\\ \underset{R_{T}\to +\infty }{\lim}h\left(r^{*}\right) & =\frac{\beta_{4}r^{*}}{\beta_{4}r^{*}+\beta_{3}\left(1-r^{*}\right)} \end{array}$$

Solving for $\lim_{R_{T}\to +\infty }g^{-1}\left(r^{*}\right)=\lim_{R_{T}\to +\infty }h\left(r^{*}\right)$ leads to the quadratic equation $A{\left(r^{*}\right)}^{2}+Br^{*}+C=0$ . The result now follows upon noting that if $\left(\alpha_{1}+\alpha_{2}+\alpha_{3}S_{T}\right)\beta_{3}>\left(\alpha_{1}+\alpha_{2}+\alpha_{4}S_{T}\right)\beta_{4}$ , then A>0 and B<0 , otherwise A<0 ; by Descartes’ rule of signs, the quadratic equation has a unique positive solution.

Corollary 1 *Consider the TCS described by models* Equations 9, 10 *and let the total* SHK *concentration* $S_{T}$ *be arbitrary but fixed. Assuming the total* RR *concentration to be very high—that is,* $R_{T}\to +\infty$ *—then if* $\beta_{3}\neq 0$ *and* $\beta_{4}=0$ *, the equilibrium point asymptotically reached by the system is* $\left(r_{\min}^{*},0\right)$ *; if* $\beta_{3}=0$ *and* $\beta_{4}\neq 0$ *, the equilibrium point is* $\left(r_{\max}^{*},1\right)$ .

Proof. Consider the scenario with $\beta_{3}\neq 0$ and $\beta_{4}=0$ and note that in this case, $s_{\max}^{*}=\frac{\beta_{2}}{\beta_{1}+\beta_{2}}$ . Taking the limit for $R_{T}\to +\infty$ of SHK nullcline (12) yields

$$s_{eq}^{*}=\underset{R_{T}\to +\infty }{\lim}\frac{\beta_{2}}{\beta_{1}+\beta_{2}+\beta_{3}R_{T}\left(1-r^{*}\right)}=0,\;s_{\min}^{*}=0.$$

Then, from RR nullcline Equation 11, we have $r_{eq}^{*}=g\left(s_{eq}^{*}\right)=r_{\min}^{*}$ ^4^. The proof for the case $\beta_{3}=0$ and $\beta_{4}\neq 0$ follows the same line and is hence omitted.

**FIGURE 4:**
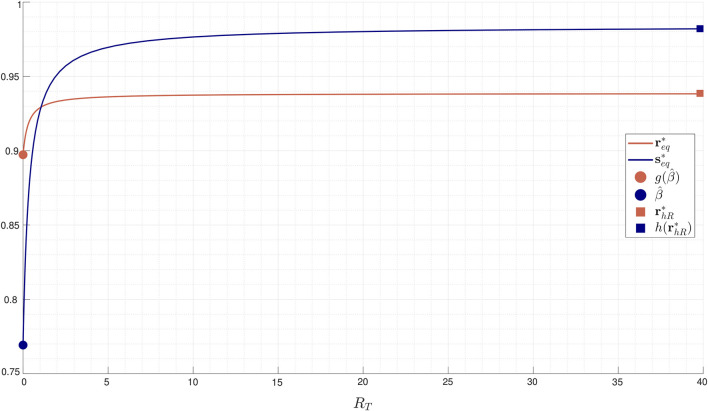
Equilibrium values $r_{eq}^{*}$ and $s_{eq}^{*}$ continuously depend on the total amount of RR protein $R_{T}$ . Parameter values: $\alpha_{1}=0.5$ , $\alpha_{2}=1$ , $\alpha_{3}=1$ , $\alpha_{4}=7$ , $\beta_{1}=0.3$ , $\beta_{2}=1$ , $\beta_{3}=1$ , $\beta_{4}=4$ , and $S_{T}=1$ .

Figure 4 reports, for an illustrative set of parameters, equilibrium values $r_{eq}^{*}$ and $s_{eq}^{*}$ as a function of $R_{T}$ .

By symmetry, analogous results on the equilibrium point hold when the SHK total amount is extremely low or extremely high— $S_{T}\to 0$ or $S_{T}\to +\infty$ .

### 3.2 Uniform monotonicity of the equilibrium with respect to $R_{T}$ and $S_{T}$

We now consider small perturbations of $R_{T}$ and $S_{T}$ concentrations and investigate their effects on the equilibrium point $\left(r_{eq}^{*},s_{eq}^{*}\right)$ .

We assume first that $S_{T}$ is constant and consider small perturbations of $R_{T}$ . The equilibrium values continuously depend on $R_{T}$ —that is, $\left(r_{eq}^{*},s_{eq}^{*}\right)=\left(g\left(s_{eq}^{*},R_{T}\right),h\left(r_{eq}^{*},R_{T}\right)\right)$ —and this dependence is quantitatively described by

$$\frac{\partial r_{eq}^{*}}{\partial R_{T}}=\frac{\partial g}{\partial s^{*}}\frac{\partial s_{eq}^{*}}{\partial R_{T}}$$ (14)

$$\frac{\partial s_{eq}^{*}}{\partial R_{T}}=\frac{\partial h}{\partial r^{*}}\frac{\partial r_{eq}^{*}}{\partial R_{T}}+\frac{\partial h}{\partial R_{T}}$$ (15)

Conversely, if we assume that total concentration $R_{T}$ is constant while $S_{T}$ slowly varies, we have

$$\frac{\partial r_{eq}^{*}}{\partial S_{T}}=\frac{\partial g}{\partial s^{*}}\frac{\partial s_{eq}^{*}}{\partial S_{T}}+\frac{\partial g}{\partial S_{T}}$$ (16)

$$\frac{\partial s_{eq}^{*}}{\partial S_{T}}=\frac{\partial h}{\partial r^{*}}\frac{\partial r_{eq}^{*}}{\partial S_{T}}$$ (17)

Putting together Equations 14–17 and solving for the variation of equilibria with respect to $R_{T}$ and $S_{T}$ , we obtain

$$\frac{\partial r_{eq}^{*}}{\partial R_{T}}=\frac{\frac{\partial h}{\partial R_{T}}\frac{\partial g}{\partial s^{*}}}{1-\frac{\partial h}{\partial r^{*}}\frac{\partial g}{\partial s^{*}}}\frac{\partial s_{eq}^{*}}{\partial R_{T}}=\frac{\frac{\partial h}{\partial R_{T}}}{1-\frac{\partial h}{\partial r^{*}}\frac{\partial g}{\partial s^{*}}}$$ (18)

$$\frac{\partial r_{eq}^{*}}{\partial S_{T}}=\frac{\frac{\partial g}{\partial S_{T}}}{1-\frac{\partial h}{\partial r^{*}}\frac{\partial g}{\partial s^{*}}}\frac{\partial s_{eq}^{*}}{\partial S_{T}}=\frac{\frac{\partial g}{\partial S_{T}}\frac{\partial h}{\partial r^{*}}}{1-\frac{\partial h}{\partial r^{*}}\frac{\partial g}{\partial s^{*}}}$$ (19)

Proposition 5 Consider the TCS described by model Equations 9, 10, and let $\left(r_{eq}^{*},s_{eq}^{*}\right)$ denote the (unique) equilibrium point of the system. The equilibrium values $r_{eq}^{*}=r_{eq}^{*}\left(R_{T},S_{T}\right)$ *and* $s_{eq}^{*}=s_{eq}^{*}\left(R_{T},S_{T}\right)$ *are:*

i) *monotonically increasing in their arguments if* $\hat{\alpha }>\bar{\beta }$ *and* $\hat{\beta }>\bar{\alpha }$ *;*
ii) *monotonically decreasing in their arguments if* $\hat{\alpha }<\bar{\beta }$ *and* $\hat{\beta }<\bar{\alpha }$ *.*

Proof. Observe that

$$\frac{\partial h}{\partial r^{*}}=\frac{R_{T}\left(\beta_{1}\beta_{4}+\beta_{4}\beta_{3}R_{T}+\beta_{3}\beta_{2}\right)}{{\left(\beta_{1}+\beta_{2}+\beta_{3}R_{T}\left(1-r^{*}\right)+\beta_{4}R_{T}r^{*}\right)}^{2}}\geq 0\;\text{for every }r^{*}\in \left[0,1\right],$$

and by symmetry, also $\frac{\partial g}{\partial s^{*}}\geq 0$ for every $s^{*}\in \left[0,1\right]$ . Moreover, recall that the function $\Delta \left(r^{*}\right)≔h\left(r^{*}\right)-g^{-1}\left(r^{*}\right)$ is such that Δ(0)>0 and Δ(1)<0 (see proof of Theorem 1), and hence at the equilibrium $\frac{\partial \Delta }{\partial r^{*}}=\frac{\partial h}{\partial r^{*}}-\frac{\partial g^{-1}}{\partial r^{*}}<0$ — $\frac{\partial h}{\partial r^{*}}<\frac{\partial g^{-1}}{\partial r^{*}}$ . This, in turn, implies that

$$0<\frac{\frac{\partial h}{\partial r^{*}}}{\frac{\partial g^{-1}}{\partial r^{*}}}=\frac{\partial h}{\partial r^{*}}\frac{\partial g}{\partial s^{*}}<1$$

Then, the sign of the partial derivatives Equations 18, 19 are solely determined by $\frac{\partial h}{\partial R_{T}}$ and $\frac{\partial g}{\partial S_{T}}$ since all other terms are always non-negative. It is a matter of computation to verify that

$$\frac{\partial h}{\partial R_{T}}=\frac{\left(\beta_{1}\beta_{4}+\beta_{2}\beta_{3}\right)r^{*}-\beta_{2}\beta_{3}}{{\left(\beta_{1}+\beta_{2}+\beta_{3}R_{T}\left(1-r^{*}\right)+\beta_{4}R_{T}r^{*}\right)}^{2}},$$

and hence at equilibrium $sign\left(\frac{\partial h}{\partial R_{T}}\right)=sign\left(r_{eq}^{*}-\bar{\beta }\right)$ . Exploiting again the symmetry of the system, we can claim that $sign\left(\frac{\partial g}{\partial S_{T}}\right)=sign\left(s_{eq}^{*}-\bar{\alpha }\right)$ . Hence, provided that $r_{eq}^{*}$ $\left(s_{eq}^{*}\right)$ is greater than $\bar{\beta }$ (respectively, $\bar{\alpha }$ ), both $r_{eq}^{*}$ and $s_{eq}^{*}$ are monotonically increasing functions of $R_{T}$ (respectively, $S_{T}$ ). Similarly, provided that $r_{eq}^{*}$ $\left(s_{eq}^{*}\right)$ is smaller than $\bar{\beta }$ (respectively, $\bar{\alpha }$ ), both $r_{eq}^{*}$ and $s_{eq}^{*}$ are monotonically decreasing functions of $R_{T}$ (respectively, $S_{T}$ ). It is clear from Figure 2 that when $\hat{\alpha }>\bar{\beta }$ and $\hat{\beta }>\bar{\alpha }$ , the equilibrium values necessarily satisfy the inequalities $r_{eq}^{*}>\bar{\beta }$ and $s_{eq}^{*}>\bar{\alpha }$ , and the thesis follows.

Remark 2 *The conditions on the system parameters provided by* proposition 5 *are sufficient (but not necessary) for uniform monotonicity of the equilibrium concerning total concentrations* $R_{T}$ *and* $S_{T}$ *. It is worth noticing that such a result is extremely powerful; its strength resides in the fact that it does not depend on the specific form of the functions* $R_{T}$ *and* $S_{T}$ *(provided they are monotone). More specifically, let* $R_{T}=f_{R}\left(u_{\mathrm{ext}}\right)$ *and* $S_{T}=f_{S}\left(u_{\mathrm{ext}}\right)$ *, where* $u_{\mathrm{ext}}$ *is an external signal and* $f_{R}$ *and* $f_{S}$ *are monotone functions. Then,* $u_{\mathrm{ext}}=f_{R}^{-1}\left(R_{T}\right)$ *, and the relationship between* $S_{T}$ *and* $R_{T}$ *is given by* $S_{T}=f_{S}◦f_{R}^{-1}\left(R_{T}\right)$ *(note that the composite function* $f_{S}◦f_{R}$ *is itself monotone).* Proposition 5 *states that if* $\hat{\alpha }>\bar{\beta }$ *and* $\hat{\beta }>\bar{\alpha }$ *, monotonicity of the equilibrium with respect to* $R_{T}$ *and* $S_{T}$ *is ensured independently on the specific form of the monotone functions* $f_{R}$ *and* $f_{S}$ *. If the previous conditions are not satisfied, uniform monotonicity is not guaranteed.*

We now focus on the case where a proportionality relationship among $R_{T}$ and $S_{T}$ can be assumed: $S_{T}=f_{S}◦f_{R}\left(R_{T}\right)=\lambda R_{T}$ . Note that this is a perfectly reasonable assumption when phosphorylated RR activates the transcription of both its gene and the gene encoding its partner SHK —see, for example, the mathematical description of the *MprA*/*MprB* two-component system adopted in (Tiwari et al., 2010).

Theorem 1 *Consider the TCS described by models* Equations 9, 10 *, and assume that total* RR *and* SHK *concentrations are related by* $S_{T}=\lambda R_{T}$ *, where* λ>0 *is a fixed (not necessarily known) proportionality coefficient. When the total* RR *concentration is extremely high—that is, for* $R_{T}\to +\infty$ *—the (unique) equilibrium point asymptotically reached by the system is*

$$\left(r_{eq}^{*},s_{eq}^{*}\right)=\left\{\begin{array}{cc} \left(0,0\right),\; & \text{if}\frac{\alpha_{3}\beta_{3}}{\alpha_{4}\beta_{4}}>1\\ \left(1,1\right),\; & \text{if}\frac{\alpha_{3}\beta_{3}}{\alpha_{4}\beta_{4}}<1 \end{array}\right.$$

Proof. Compute the limit for $R_{T}\to +\infty$ of RR and SHK nullclines Equations 11, 12:

$$r^{*}=\underset{R_{T}\to +\infty }{\lim}g\left(s^{*},\lambda R_{T}\right)=\frac{\alpha_{4}s^{*}}{\alpha_{3}+\left(\alpha_{4}-\alpha_{3}\right)s^{*}},\text{for every }\lambda >0$$ (20)

$$s^{*}=\underset{R_{T}\to +\infty }{\lim}h\left(r^{*},R_{T}\right)=\frac{\beta_{4}r^{*}}{\beta_{3}+\left(\beta_{4}-\beta_{3}\right)r^{*}}$$ (21)

From expression Equation 21, it is easy to obtain

$$r^{*}=h^{-1}\left(s^{*}\right)=\frac{\beta_{3}s^{*}}{\beta_{4}+\left(\beta_{3}-\beta_{4}\right)s^{*}}$$

Substituting the previous expression into Equation 20 and solving for $s^{*}$ yields the following quadratic equation:

$${\left(s^{*}\right)}^{2}\left\{\left(\alpha_{4}\beta_{4}-\alpha_{3}\beta_{3}\right)s^{*}+\left(\alpha_{3}\beta_{3}-\alpha_{4}\beta_{4}\right)\right\}=0$$

Then, the only two possible equilibrium points are $\left(r_{eq}^{*},s_{eq}^{*}\right)=\left(0,0\right)$ and $\left(r_{eq}^{*},s_{eq}^{*}\right)=\left(1,1\right)$ . To determine which is the right solution, we need to resort to the intersection condition $\frac{\partial h}{\partial r^{*}}\frac{\partial g}{\partial s^{*}}<1$ (see the proof of Proposition 5). Indeed, it is straightforward to verify that

$$\begin{array}{ll} \underset{R_{T}\to +\infty }{\lim}\frac{\partial h}{\partial r^{*}} & =\frac{\beta_{3}\beta_{4}}{{\left(\beta_{3}+\left(\beta_{4}-\beta_{3}\right)r^{*}\right)}^{2}}\\ \underset{R_{T}\to +\infty }{\lim}\frac{\partial g}{\partial s^{*}} & =\frac{\alpha_{3}\alpha_{4}}{{\left(\alpha_{3}+\left(\alpha_{4}-\alpha_{3}\right)s^{*}\right)}^{2}}, \end{array}$$

and hence

$$\begin{array}{ll} \frac{\partial h}{\partial r^{*}}\frac{\partial g}{\partial s^{*}}{|}_{\left(r^{*},s^{*}\right)=\left(0,0\right)} & =\frac{\beta_{4}}{\beta_{3}}\frac{\alpha_{4}}{\alpha_{3}}\\ \frac{\partial h}{\partial r^{*}}\frac{\partial g}{\partial s^{*}}{|}_{\left(r^{*},s^{*}\right)=\left(1,1\right)} & =\frac{\beta_{3}}{\beta_{4}}\frac{\alpha_{3}}{\alpha_{4}}, \end{array}$$

which uniquely determines the limiting equilibrium pair once the quantity $\frac{\beta_{4}}{\beta_{3}}\frac{\alpha_{4}}{\alpha_{3}}$ is known.

Remark 3 *The previous result does not require knowledge of the value assumed by the proportionality coefficient* λ *; we just need to know that a proportionality coefficient continuously relates* $R_{T}$ *and* $S_{T}$

## 4 Absolute concentrations

We have thus far analyzed the properties (asymptotic behavior and monotonicity) of *relative* concentrations: of the ratio between phosphorylated and unphosphorylated protein concentrations. A fundamental and crucial point is that these properties do not necessarily hold for absolute concentrations too: the fact that the relative concentration $r_{eq}^{*}$ tending to 0 does not imply that absolute concentration $r_{eq}^{*}$ tends to 0; similarly, uniform monotonicity of $r_{eq}^{*}$ for $R_{T}$ does not imply uniform monotonicity of $r^{*}$ to $R_{T}$ . To understand this point, note that the relative concentration $r_{eq}^{*}$ tends to 0 when total RR concentration asymptotically grows to infinity (i.e., $R_{T}\to +\infty$ ) and $r^{*}$ asymptotically approaches a given saturation level $r_{eq}^{*}\neq 0$ . Regarding monotonicity, since $r^{*}=r^{*}R_{T}$ , it holds that

$$\frac{\partial r^{*}}{\partial R_{T}}=\frac{\partial r^{*}}{\partial R_{T}}R_{T}+r^{*}$$

It is clear that if $r_{eq}^{*}$ is a monotonically increasing function of $R_{T}$ (namely, $\frac{\partial r^{*}}{\partial R_{T}}>0$ ), so is $r_{eq}^{*}$ . On the contrary, if $r_{eq}^{*}$ is a monotonically decreasing function of $R_{T}$ , and hence $\frac{\partial r^{*}}{\partial R_{T}}<0$ ; monotonicity of $r_{eq}^{*}$ with respect to $R_{T}$ is not guaranteed.

In the following, we analyze the asymptotic behavior of absolute concentrations $r_{eq}^{*}$ and $s_{eq}^{*}$ when $R_{T}$ grows to infinity, under the assumption that RR and SHK total concentrations are linearly related with the proportionality coefficient λ — $S_{T}=\lambda R_{T}$ .

Theorem 2 *Consider the TCS described by models* Equations 9, 10 *and assume that the total* RR *and* SHK *concentrations are linearly related by* $S_{T}=\lambda R_{T}$ *, where* λ>0 *is a fixed proportionality coefficient. When total* RR *concentration is sufficiently high—that is, for* $R_{T}\to +\infty$ *,* RR *and* SHK *—then absolute concentrations asymptotically approach the equilibrium values:*

$$\begin{array}{ll} r_{eq}^{*} & =\frac{\alpha_{2}\beta_{3}+\lambda \alpha_{4}\beta_{2}}{\lambda \left(\alpha_{3}\beta_{3}-\alpha_{4}\beta_{4}\right)}\\ s_{eq}^{*} & =\frac{\alpha_{2}\beta_{4}+\lambda \alpha_{3}\beta_{2}}{\alpha_{3}\beta_{3}-\alpha_{4}\beta_{4}}, \end{array}$$

respectively.

Proof. We claim that for a sufficiently high $R_{T}$ , absolute equilibrium concentrations $r_{eq}^{*}$ and $s_{eq}^{*}$ asymptotically approach saturation levels ρ and σ :

$$\begin{array}{ll} \underset{R_{T}\to +\infty }{\lim}\left\{r_{eq}^{*}\left(R_{T},\lambda R_{T}\right)\cdot R_{T}\right\} & =\rho ,\\ \underset{R_{T}\to +\infty }{\lim}\left\{s_{eq}^{*}\left(R_{T},\lambda R_{T}\right)\cdot \lambda R_{T}\right\} & =\sigma \end{array}$$

We now seek to determine the values ρ and σ . First, we note that

$$\begin{array}{ll} \rho & =\underset{R_{T}\to +\infty }{\lim}g\left(s_{eq}^{*}\left(R_{T},\lambda R_{T}\right),\lambda R_{T}\right)\cdot R_{T}\\ & =\underset{R_{T}\to +\infty }{\lim}\frac{\alpha_{4}\lambda R_{T}s_{eq}^{*}\left(R_{T},\lambda R_{T}\right)+\alpha_{2}}{\alpha_{1}+\alpha_{2}+\alpha_{3}\lambda R_{T}\left(1-s_{eq}^{*}\left(R_{T},\lambda R_{T}\right)\right)+\alpha_{4}\lambda R_{T}s_{eq}^{*}\left(R_{T},\lambda R_{T}\right)}\cdot R_{T}\\ & =\underset{R_{T}\to +\infty }{\lim}\frac{\alpha_{4}\sigma +\alpha_{2}}{\alpha_{1}+\alpha_{2}+\alpha_{3}\lambda R_{T}+\left(\alpha_{4}-\alpha_{3}\right)\sigma }\cdot R_{T}\\ & =\frac{\alpha_{4}\sigma +\alpha_{2}}{\lambda \alpha_{3}} \end{array}$$

Analogously, the limit of $s_{eq}^{*}$ for $R_{T}\to +\infty$ can be computed as

$$\begin{array}{ll} \sigma & =\underset{R_{T}\to +\infty }{\lim}h\left(r_{eq}^{*}\left(R_{T},\lambda R_{T}\right),\lambda R_{T}\right)\cdot \lambda R_{T}\\ & =\underset{R_{T}\to +\infty }{\lim}\frac{\beta_{4}R_{T}r_{eq}^{*}\left(R_{T},\lambda R_{T}\right)+\beta_{2}}{\beta_{1}+\beta_{2}+\beta_{3}R_{T}\left(1-r_{eq}^{*}\left(R_{T},\lambda R_{T}\right)\right)+\beta_{4}R_{T}r_{eq}^{*}\left(R_{T},\lambda R_{T}\right)}\cdot \lambda R_{T}\\ & =\underset{R_{T}\to +\infty }{\lim}\frac{\beta_{4}\rho +\beta_{2}}{\beta_{1}+\beta_{2}+\beta_{3}R_{T}+\left(\beta_{4}-\beta_{3}\right)\rho }\cdot \lambda R_{T}\\ & =\frac{\left(\beta_{4}\rho +\beta_{2}\right)\lambda }{\beta_{3}} \end{array}$$

Therefore, we need to solve the linear system:

$$\left\{\begin{array}{c} \rho \lambda \alpha_{3}=\alpha_{4}\sigma +\alpha_{2}\;\\ \sigma \beta_{3}=\left(\beta_{4}\rho +\beta_{2}\right)\lambda \; \end{array}\right.$$

Solving for ρ and σ yields

$$\rho =\frac{\alpha_{2}\beta_{3}+\lambda \alpha_{4}\beta_{2}}{\lambda \left(\alpha_{3}\beta_{3}-\alpha_{4}\beta_{4}\right)},\;\sigma =\frac{\alpha_{2}\beta_{4}+\lambda \alpha_{3}\beta_{2}}{\alpha_{3}\beta_{3}-\alpha_{4}\beta_{4}},$$

Thus, the proof is concluded.

It follows from Theorem 2 that for sufficiently high $R_{T}$ , while the amount of phosphorylated SHK increases with λ , the amount of phosphorylated RR is a decreasing function of λ , such that

$$r_{eq}^{*}=\frac{\frac{1}{\lambda }\alpha_{2}\beta_{3}+\alpha_{4}\beta_{2}}{\alpha_{3}\beta_{3}-\alpha_{4}\beta_{4}}$$ (22)

## 5 Discussion

A distinguishing feature of the proposed TCS mathematical model is that it accounts for a variety of reactions, including RR phosphorylation and dephosphorylation through external (exogenous) pathways, SHK autophosphorylation and autodephosphorylation, RR phosphorylation via phosphotransfer from SHK , and RR dephosphorylation via SHK . Of course, by setting 0 for one or more parameters, the model can be tailored to specific two-component systems (TCSs) and/or situations in which some of the previous reactions are negligible.

One of the best characterized examples of TCS is the *EnvZ*/*OmpR* system in *Escherichia coli*, which responds to changes in environmental osmolality by regulating the expression of the outer membrane porins *OmpF* and *OmpC*. As in many TCSs, *EnvZ* is a bifunctional sensor histidine kinase, meaning that it phosphorylates and dephosphorylates the response regulator *OmpR*. Batchelor and Goulian (2003) proposed a mathematical model of the *EnvZ*/*OmpR* TCS and experimentally tested the model’s predictions. Their main finding was that for sufficiently high amounts of *OmpR*, when total *EnvZ* in the cell is much less abundant than total *OmpR* ^5^, the steady-state level of phosphorylated *OmpR* is robust (insensitive) to fluctuations in *EnvZ* and *OmpR* concentrations. This model accounts for the autokinase, phosphotransfer, and phosphatase activities of *EnvZ* and neglects the exogenous phosphorylation and dephosphorylation of *OmpR*. Casting such a scenario into our mathematical framework means setting $\alpha_{2}$ and $\beta_{4}$ to 0. Theorem 2 then implies that the equilibrium absolute concentration for *OmpR* is given by $r_{eq}^{*}=\frac{\alpha_{4}\beta_{2}}{\alpha_{3}\beta_{3}}$ , and hence, consistent with Batchelor and Goulian (2003), does not depend on *EnvZ* total concentration. However, our model shows that if an exogenous RR phosphorylation flux is present ( $\alpha_{2}\neq 0$ ), the previous result fails; when an external pathway for *OmpR* phosphorylation is present, the steady-state concentration of phosphorylated *OmpR* is (higher and) decreasing with λ (see Equation 22). Notably, Batchelor and Goulian (2003) predicted, via theoretical analysis and experimental verification with fluorescent reporter strains, that when condition $S_{T}\ll R_{T}$ does not hold, the steady-state value of *OmpR-P* decreases with increasing total *EnvZ* concentration. This is consistent with our theoretical results, which also shed light on the role of an *EnvZ*-independent mechanism for *OmpR* phosphorylation.

Furthermore, our analysis allows the characterization of the steady-state concentration of the histidine kinase: $s_{eq}^{*}=\lambda \frac{\beta_{2}}{\beta_{3}}$ (recall that $\beta_{4}=0$ ). As expected, our model predicts that the amount of phosphorylated *EnvZ* increases with more vigorous autokinase activity $\left(\beta_{2}\right)$ and decreases with stronger phosphotransfer activity of the histidine kinase $\left(\beta_{3}\right)$ .

Finally, while our analysis demonstrates the existence of a single robust equilibrium of the system (Theorem 1), it is instructive to consider the possibility of using such a building block as part of a closed-loop system with positive retroactivity, which could lead to oscillatory or bistable behaviors (Igoshin et al., 2008; Zorzan et al., 2021; Tiwari et al., 2010).

### 5.1 Phosphotransfer and reverse phosphotransfer reactions

Bifunctional sensor histidine kinase exerts both positive and negative control through SHK phosphotransfer and phosphatase activity, respectively. While the biochemical reactions underlying SHK kinase activity are reasonably well understood, the mechanisms of phosphatase activity represent a long-standing question, the investigation of which has led to the formulation of multiple hypotheses (see Huynh and Stewart, 2011 for an overview). An early hypothesis, first proposed by Dutta and Inouye (1996), identified reverse transfer of the phosphoryl group from phosphorylated RR to SHK as a potential RR dephosphorylation mechanism. Such a hypothesis was prompted by experimental results conducted on *EnvZ*/*OmpR* system in *E. coli* (Dutta and Inouye, 1996; Zhu et al., 2000), showing that reverse transfer of the phosphoryl group from *OmpR-P* to *EnvZ* was detected in the early period of the phosphatase reaction with domain A of *EnvZ*—specifically with the *EnvZ* ${\text{kinase}}^{-}$ ${\text{phosphatase}}^{+}$ mutant (*EnvZ.N347D*), and, under certain conditions, with the wild-type *EnvZ*.

Even if later experiments invalidated the reverse phosphotransfer model (Hsing and Silhavy, 1997), it is universally recognized that reverse phosphotransfer can occur under certain conditions. As pointed out by Gao and Stock (2009), multiple mechanisms may have evolved for phosphatase activities, and individual histidine kinases may utilize different regulatory strategies. We now aim to theoretically investigate a scenario in which both direct and reverse phosphotransfer reactions occur, and a distinct phosphatase activity of the sensor histidine is present.

Since the kinase activity of SHK takes the form of a phosphotransfer reaction (by which a phosphoryl group is transferred from phosphorylated SHK to RR ), reaction rates $\alpha_{4}$ and $\beta_{3}$ are actually equal— $\alpha_{4}=\beta_{3}$ . We first assume that only SHK exhibits phosphotransfer activity ( $\beta_{4}=0$ ), and we rename $\alpha_{3}$ as $\alpha_{3}^{p}$ , where superscript p stands for “phosphatase activity” (of the SHK ). It follows from Theorem 2 that when total RR concentration is sufficiently high, steady-state absolute concentrations are given by $r_{eq}^{*}=\frac{\frac{1}{\lambda }\alpha_{2}+\beta_{2}}{\alpha_{3}^{p}}$ and $s_{eq}^{*}=\lambda \frac{\beta_{2}}{\beta_{3}}$ .

When reverse phosphotransfer from phosphorylated RR to SHK occurs, the reaction rate $\beta_{4}$ is non-zero and $\alpha_{3}=\alpha_{3}^{p}+\alpha_{3}^{rt}$ , with $\alpha_{3}^{rt}=\beta_{4}$ (where superscript rt stands for “reverse phosphotransfer”). Then, recalling that $\alpha_{4}=\beta_{3}$ , Theorem 2 yields

$$r_{eq}^{*}=\frac{\frac{1}{\lambda }\alpha_{2}+\beta_{2}}{\alpha_{3}^{p}}\;\text{ and }\;s_{eq}^{*}=\lambda \frac{\beta_{2}}{\beta_{3}}+\frac{\alpha_{2}\beta_{4}+\lambda \alpha_{3}^{rt}\beta_{2}}{\alpha_{3}^{p}\beta_{3}}=\lambda \frac{\beta_{2}}{\beta_{3}}+\frac{\alpha_{3}^{rt}\left(\alpha_{2}+\lambda \beta_{2}\right)}{\alpha_{3}^{p}\beta_{3}}$$

This indicates that, even if reverse phosphotransfer occurs, the absolute concentration of phosphorylated RR remains unchanged. While this may seem contradictory at first, it is easily explained by noting that reverse phosphotransfer from phosphorylated RR to SHK is exactly compensated by the increased direct phosphotransfer from phosphorylated SHK to RR . On the contrary, when the reverse phosphotransfer reaction occurs, our analysis shows that the absolute concentration of SHK increases and that such an increase is larger for higher values of the reverse phosphotransfer rate (bigger $\alpha_{3}^{rt}$ ) and/or for larger amounts of total SHK concentration (bigger λ ).

This study’s main findings are summarized here in comparison with the literature.

## 6 Conclusion

We here developed a generalized mathematical model for bacterial two-component signaling systems that integrates canonical phosphorylation cycles, bifunctional enzymatic activities, transcriptional feedback, and potential auxiliary interactions. Through systems-level analysis, we elucidated how network architecture and parameter regimes shape key dynamic properties and robustness.

Our modeling framework provides a predictive foundation for interpreting experimental dynamics, as illustrated for the EnvZ/OmpR system, and for guiding the rational design of synthetic signaling circuits. We demonstrated that the bifunctionality of the sensor histidine kinase, multi-step phosphorelays, and transcriptional feedback, which are incorporated into the model, enable rich behaviors that allow TCSs to precisely tune cellular responses to diverse environmental stimuli.

Notably, we derived analytical conditions in Propositions 3, Propositions 4, Propositions 5 and Theorem 1 under which the steady-state levels of phosphorylated proteins exhibit input–output robustness, overshoot, or bistability. We also characterized in Sections 3–4 how the equilibrium phosphorylation levels depend on the absolute and relative abundances of the two components. These insights are critical for understanding natural mechanisms of bacterial adaptation and for forward-engineering synthetic gene circuits with prescribed dynamics.

By combining the mechanistic modeling framework with systems analysis techniques, such as nullcline analysis, this study provides a unified perspective on the structural design principles that underlie the remarkable versatility of two-component signal transduction. The proposed generalized model lays a theoretical foundation for further experimental investigations, such as exploring reverse phosphotransfer mechanisms, and establishes a framework for rationally harnessing two-component systems in synthetic biology applications.

## Data Availability

The original contributions presented in the study are included in the article/supplementary material; further inquiries can be directed to the corresponding author.

## Footnotes

1 Actually, in (Tiwari et al., 2010) production of RR and SHK is described by the summation of two activating Hill functions. As explained later, since we are focusing on the functioning of the Two-Component System, the separation of time scales allows us to assume constant production rates.

2 Since every limit cycle is a closed orbit, ruling out the existence of closed orbits automatically excludes the existence of limit cycles.

3 An equivalent way to see that $s_{eq}^{*}=\hat{\beta }$ when $R_{T}\to 0$ is noticing that in this case both $s_{\min}^{*}$ and $s_{\max}^{*}$ tend to $\beta_{2}/\left(\beta_{1}+\beta_{2}\right)=\hat{\beta }$ , and hence the subregion $C_{eq}$ reduces to a line.

4 Alternatively, the result directly follows from Proposition 4 with $\beta_{3}=0$ .

5 As reported, for instance, in (Hsing and Silhavy, 1997), *in vivo OmpR* is nearly 100-fold more abundant than *EnvZ*.
